# A Malicious Behavior Awareness and Defense Countermeasure Based on LoRaWAN Protocol

**DOI:** 10.3390/s19235122

**Published:** 2019-11-22

**Authors:** Shu-Yang Gao, Xiao-Hong Li, Mao-De Ma

**Affiliations:** 1 College of Intelligence and Computing, Tianjin University, Tianjin 300350, China; gaoshuyang@tju.edu.cn; 2 Tianjin Key Laboratory of Advanced Networking, Tianjin 300350, China; 3 School of Electrical and Electronic Engineering, Nanyang Technological University, Singapore 639798, Singapore; emdma@ntu.edu.sg

**Keywords:** awareness, defense, LoRaWAN, replay attack

## Abstract

Low power wide area network (LoRaWAN) protocol has been widely used in various fields. With its rapid development, security issues about the awareness and defense against malicious events in the Internet of Things must be taken seriously. Eavesdroppers can exploit the shortcomings of the specification and the limited consumption performance of devices to carry out security attacks such as replay attacks. In the process of the over-the-air-activation (OTAA) for LoRa nodes, attackers can modify the data because the data is transmitted in plain text. If the user’s root key is leaked, the wireless sensor network will not be able to prevent malicious nodes from joining the network. To solve this security flaw in LoRaWAN, we propose a countermeasure called Secure-Packet-Transmission scheme (SPT) which works based on the LoRaWAN standard v1.1 to prevent replay attacks when an attacker has obtained the root key. The proposed scheme redefines the format of join-request packet, add the new One Time Password (OTP) encrypted method and changes the transmission strategy in OTAA between LoRa nodes and network server. The security evaluation by using the Burrows-Abadi-Needham logic (BAN Logic) and the Scyther shows that the security goal can be achieved. This paper also conducts extensive experiments by simulations and a testbed to perform feasibility and performance analysis. All results demonstrate that SPT is lightweight, efficient and able to defend against malicious behavior.

## 1. Introduction

The Long Range Wide Area Network (LoRaWAN) protocol [1] has been widely used in various industrial Internet-of-Things (IoT) scenarios such as smart agriculture, smart fire control system, smart buildings, and so on. It is a new type of wireless communication protocol designed to support low power and long range wireless communications at a low data transmission rate dedicated to various IoT applications. The network is deployed in a star topology [2] with a GateWay (GW) to transfer messages between LoRa node and Network Server (NS). It can be used flexibly in regional, national or global networks. With the help of open source LoRaWAN servers, users can easily build their own private services. That is the reason why the protocol must guarantee the reliability of the data in the sensor network. Since LoRaWAN specifies that the communication frequency in a region is known as long as the node is set to the specified frequency so that the data at this frequency can be monitored. Because of this, the communication data of the normal node is inevitably monitored [3]. Under this circumstance, the data itself needs to have the ability to prevent decryption after it has been eavesdropping.

As disclosed in [4], the replay attack is the most popular security attack threat to LoRaWAN. Although many recent solutions have been proposed to address the security issues in the LoRaWAN v1.1 protocol, they have not been aware of the impact of the vulnerability of the key management in the LoRaWAN networks. In fact, there is no protection for the root key in the LoRaWAN specification. A lot of works have studied the way to improve the security functionality. Some of them try to overcome the security vulnerabilities in LoRaWAN. The solutions in [5,6,7,8] attempt to solve security flaws. A new protocol based on AES128 has been proposed in [5]. It redefines the process when an ED joins the network. The solutions in [7,8] upgrade diversity of the keys to enhancing communication security when LoRaWAN is working. Their research can enhance the security functionality of the protocol. But they cannot work effectively if the root key is obtained by the attacker.

The root key is generally kept by the user, but this does not mean that all users can keep their sensitive data safe. According to the latest Open Web Application Security Project (OWASP) report [9], sensitive data leaks have occurred from time to time. For example, The search software can be installed on a personal computer or a server. Such software has the ability to connect remotely, but some users do not set a password when they turn on this feature. Hackers can rely on Google hack technology to search the IP of these computers and directly download data from their computers. Once the database is hacked, the sensitive data from the database will be completely leaked. To address it, we have our motivation to design the SPT which can provide full protection for the system based on LoRaWAN even the root keys have been compromised. The format of the join request sent by a LoRa node has been redesigned and dynamically encrypted. In the transmission phase, by originally sending the redesigned message, the attacker could be deceived. With this design, SPT has the ability to detect such behaviors like replay attack and defend against it.

In this paper, our proposed solution holds the following important contributions:

- We propose a New One-Time-Password (OTP) method that can be used in LoRaWAN protocol. This method can effectively encrypt the plain text (join request data). Through theoretical analysis, we prove that this encryption algorithm is safe and reliable.
- Original join-request message has been redefined as New-join-request (Njr). Based on the characteristics of the original join request message, a new dynamic coding technique is proposed to generate Njr. If Njr is intercepted, it is almost impossible for the attacker to perform correct decoding.
- We used Ban Logic and Scyther for formal verification. The results of formal verification prove that the theory of the protocol is safe. We also validated our scheme by using the Omnet++ following the way in [10]. The simulation results show the feasibility and robustness of the SPT.
- Our countermeasure has been implemented on the SM1276 node. We design a testbed to verify the effectiveness and security of the method at the same time.

To the best of our knowledge, this is the first study to address the malicious behavior issue in root key management. The remainder of this paper is organized as follows. The necessary technical preliminaries are discussed in Section 2. The original System Model and our proposed model are described Section 3. Security analysis and Formal verification on Scyther have shown in Section 4. Section 5 demonstrates the experiments’ design and the results with a discussion. Finally, the conclusion and related work are reached in Section 6 and Section 7.

## 2. Background

In this section, we briefly describe some basic concepts and technical preliminaries used in the design of our proposed protocols, including the security mechanism of the OTAA, the One Time Password, the Adaptive Data Rate (ADR) algorithm, and the Burrows-Abadi-Needham logic (BAN logic).

### 2.1. The Security Mechanism of the OTAA

**Figure 1:**
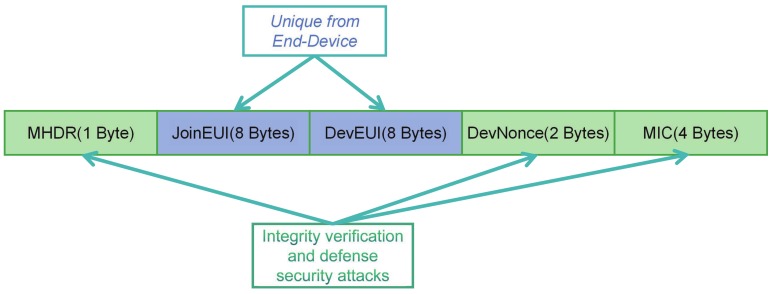
Join-Request data Structure.

Figure 1 has shown the structure of the join request. The meaning of each field is described below.

1. In join-request, MHDR represents the data type;
2. JoinEUI and DevEUI contain the basic information of a node, which is unique;
3. DevNonce is used to record the number for joining time.Changing the DevNonce and MIC is the countermeasure of defending against replay attacks.
4. The MIC is calculated by AES128 (NwkKey, MHDR|JoinEUI|DEVEUI|DevNonce). We can see the importance of the NwkKey (the root key) is irreplaceable from this Equation. Without the root key, any process that requires encryption cannot be completed. Therefore, if the root key cannot be properly kept, all security measures may fail.

### 2.2. One Time Password

One Time Password (OTP) [11] is also known as one-time pin or dynamic password which is valid for only one access session or transaction. It is a password contrast to the static passwords. The operation of logging in with the SMS verification code is one of the application scenarios of the OTP. The most important advantage for this method is that they are not vulnerable to replay attack. In our method, as an implementation of the stream cipher, the core encryption method formula is XOR-bitwise. Suppose *D* represents one original data and *K* represents data for XOR operations. After two successive XOR or XNOR operations, the value of *D* will not change.

### 2.3. ADR Algorithm

Adaptive Data Rate (ADR) algorithm is designed to extend the battery life of LoRa node. The ADR mechanism runs asynchronously at the LoRa node and at the network server. During normal communication, the node relies on adjusting the Spreading Factor (SF) to guarantee the Delivery ratio. The value of SF is about high, and the stability of the data sent by the node is higher, but the corresponding power consumption is also larger. The ADR algorithm lets the node to send the SF value to the server, relying on the server to adjust the SF value of the node to a moderate range. This adjustment ensures both the Delivery ratio and energy savings. Most of the complexity in ADR is assigned to the network server, to keep the nodes as simple as possible. The ADR algorithm on the node which can be called ARD-NODE is specified by the LoRa Alliance, whereas the algorithm can be called ADR-NET on the network server is defined by the network operator. Slabicki has improved on these two algorithms. They propose a simple modification wherein the max operator is replaced with the average function. They called it *ADR+*. Such a change like this can solve the problem of high variability in fast-fading conditions. It can ensure the data rate and save power consumption.

### 2.4. BAN Logic

The BAN logic can analyze authentication protocols. Based on logical rules, it is able to determine whether the exchanged information is trustworthy against the malicious nodes. It takes four steps to analyze a protocol: (1) Build the idealize the protocol model; (2) Make the initial assumptions; (3) Set the specific test goals; (4) Using the initial hypotheses and logical rules to execute the formal analysis. The notations used in the BAN logic are as follows:

- P∣≡X: Principal *P* believes a statement *X*, or *P* is entitled to believe *X*.
- #(X): Formula *X* is fresh.
- P⇒X: *P* has jurisdiction over a statement *X*.
- P⊲X: *P* sees *X*, the principal *P* received the message containing *X*, and *P* is able to read or repeat *X*.
- P∣∼X: *P* once said *X*, the principal *P* sent a message containing *X* at some time.
- (X,Y): The formula X or Y is one part of formula (X,Y).
- ${\left\{X\right\}}_{K}$: The formula *X* is encrypted using the key *K*.

### 2.5. Abbreviation

For clarity of presentation, the notations used throughout the paper are introduced in an abbreviation Table 1.

## 3. System Model

### 3.1. The Original System Model of OTAA

**Figure 2:**
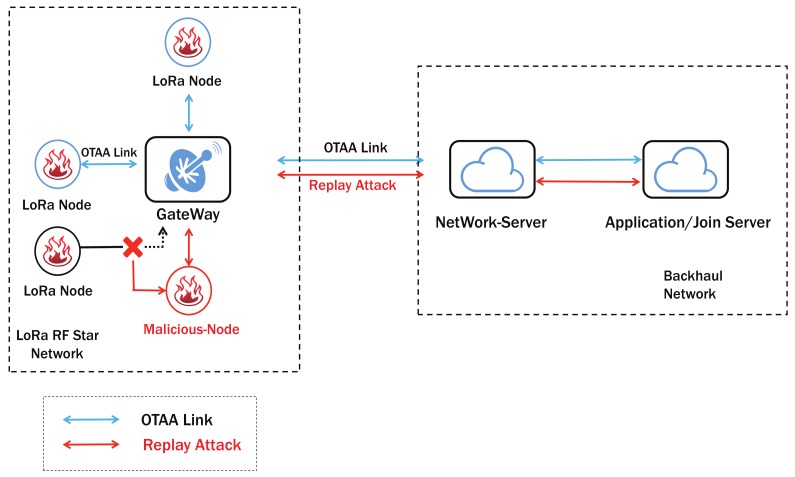
Over-the-air-activation (OTAA) Process Model.

The Over the Air Activation (OTAA) provides a flexible way of establishing session keys by root keys with nodes and servers. LoRa Node transmit its join request message at the beginning of this procedure. This message will be transformed by a GW to the NS. After its validate process, NS will send join accept back to node. The whole process is shown as blue link in Figure 2.

### 3.2. Attack Model in OTAA

The user’s node is in the normal OTAA link, but the data used for accessing the network can be intercepted directly by the attacker when they are on the same channel which cannot be avoided. When the user’s node goes off-line, the attacker can launch a replay attack like the red link in Figure 2. It is assumed that the root key data held by the user of the black LoRa Node has been leaked. After the hacker locates the black node off-line, the root key is used to immediately copy the incoming request data sent by the black node. After receiving such data, the server considers it as a request data from a normal node. After the server returns the join accept data that is allowed to join the network, the hacker’s node can join the current network after receiving the join accept data.

Once the attack is successful, the LoRa node can be replaced. After the attacker’s node completes the joining process, the user’s node will never be able to rejoin the network and the server cannot distinguish the malicious node. This will have extremely serious consequences. For example, a user’s node needs to periodically send temperature data from the room to the server. However, after the user’s node is replaced by a malicious node, the attacker will send abnormal temperature data to the server, causing the server to mistakenly think that the local temperature is abnormal, thus making an error indication.

### 3.3. Security Requirements

Under this circumstance, the original defense mechanism inside OTAA has expired. To solve this issue, our new security mechanism requires the following requirements:

1. A new authentication mechanism to ensure that the server can identify whether the data is from a malicious node.
2. This mechanism is not only effective in this case, but can directly replace the original defense mechanism to protect LoRaWAN’s network security.
3. The power consumption of each node using the new protection mechanism cannot be increased.

### 3.4. The Proposed System

**Figure 3:**
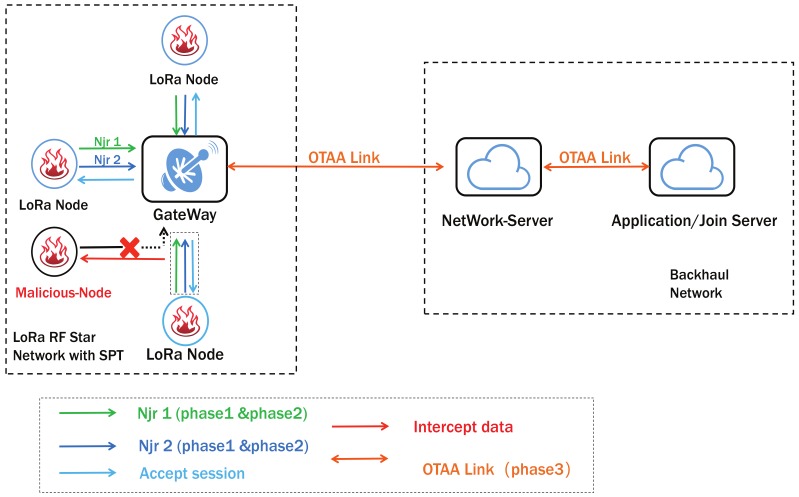
The Secure-Packet-Transmission (SPT) System.

Figure 3 has shown our SPT System. It can be seen that each LoRa Node adopts a policy of sending payloads to the Server in a packet. From this system, the blue link is the original OTAA communication, the difference is the Njr1 and Njr2 and how does a node send them to NS. It is consists of three phases: Payload Re-Encoding, Assemble Join requests, Server validation and Node Activation. The method of how to use the ADR algorithm to reduce power consumption has been discussed after our method. 

***Phase I: Payload encryption in LoRa node***

The payload we have mentioned is the jr. The original jr is a plain text transmission. It can be easily imitated after the attacker gains the root key. We use the random number generator to randomly take a single value from the root key. Let DevEUI1 = AppKey[pos1] ⊕ DevEUI, JoinEUI1 = AppKey[pos1] ⊕ JoinEUI. In addition, we also need to encrypt the MIC. Let a variable called value = DevNonce|JoinEUI|DevEUI. The Value uses the previously calculated pos1 to select a bit as the value for encryption. Let Encode-Mic=Mic ⊕ Value. We named the encrypted result as en-MIC.

***Phase II: Assemble Join requests***

**Figure 4:**
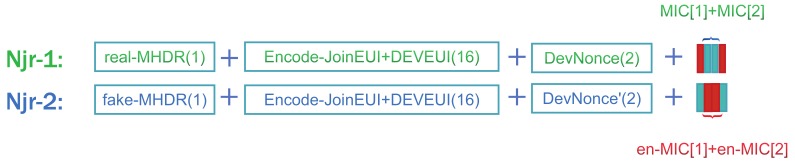
Packet Join-requests.

After the encoding process, the contents of the MIC are dispersed in Njr1 and Njr2. DevNonce in Njr2 is larger than DevNonce in Njr1. In the attacker’s view, Njr1 and Njr2 are two different payloads from one LoRa node. But in fact, they only contain the information for one original payload. The method of recombination is divided into the following steps:

1. Combine the first and fourth positions of the MIC and the second and third positions of the En-Mic together as MIC1. MIC2 is the remaining four bytes of data to combine.
2. Generate Fake-MHDR by storing the *pos* value.
3. According to the order in Figure 4 to assemble Njr1 and Njr2.

GW in the SPT still encapsulates the data as specified in OTAA. It will receive two join-request messages from node under one channel. Two Njrs will be forwarded to NS along with a JSON data. 

***Phase III: Network Server validation and LoRa Node Activation***

The verification steps in NS are required for each set of payload:

**Step 1.**  Check if the DevNnonce2 is equal to DevNonce1 plus one;
**Step 2.**  Restore JoinEUI and DevEUI by the value from fake-MHDR;
**Step 3.**  Verify the relationship between Encode-MIC and MIC. The join accept message can be generated by a network server only through all the above steps. After this part, all communication processes will be consistent with the original LoRaWAN. Otherwise NS will refuse to reply.

**Figure 5:**
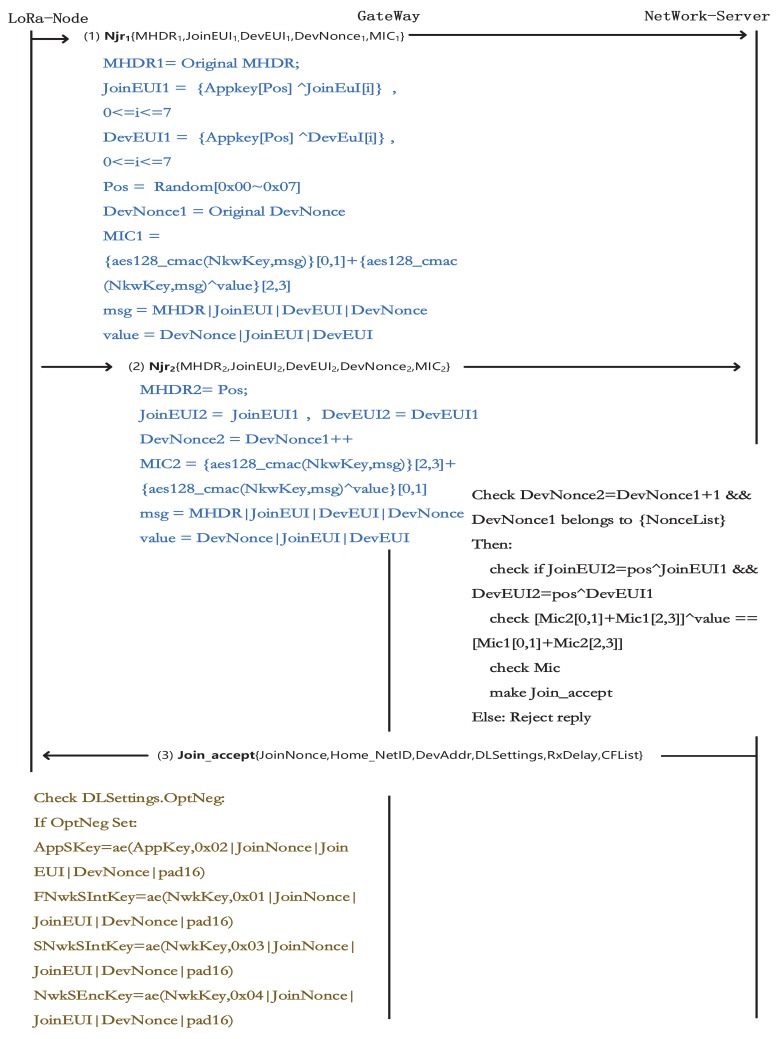
The sequence chart of SPT.

This verification process enhances the server’s ability to defend against malicious attacks. After receiving the join accept sent by the NS, the node will calculate the dynamic key according to the original protocol. Such as AppSkey, FNwkSIntKey, SNwkSIntKey, NwkSEncKey. Figure 5 shows all the interaction process and data generation methods for the three phases. It can be seen that after the original Join request data has been encrypted with existing information and the server can restore the encrypted data to its original state.

### 3.5. The Advantage for Encryption Strategy

System with SPT can detect and defend against the replay attack behavior in three aspects:

1. The attacker did not modify the unique part.
2. The attacker cannot directly derive the specific meaning of En-Mic because it is a randomly inserted noise data. So the only thing for attacker can do is to modify the MIC part directly.
3. The attacker did not use the correct number for the encoding operation.

**Figure 6:**
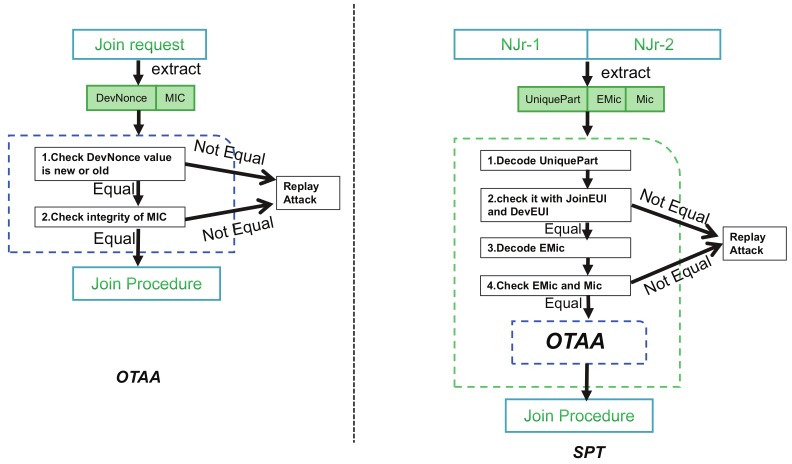
Comparison between Replay attack detection process.

Figure 6 depicts the process of SPT and OTAA for replay attack detection. OTAA relies on Mic integrity detection and DevNonce value detection to determine whether the Join request was sent by a normal node. The reason for an attacker can launch the replay attack is because DevEUI and JoinEUI are unique in join request. An attacker can attack a particular node based on the unique bytes. From Figure 4 we can see that the Encode-JoinEUI and Encode-DevEUI of each two Njr are the same. Assume that the node successfully sends the network after sending two groups of Njr in the process of SPT and all of the data is intercepted by the attacker. The attacker will consider as two Join-requests data from node A and another two Join-requests data from node B. Even if the attacker knows the principle of SPT interception, it cannot locate the node through the intercepted data. So the user’s security can be fully guaranteed. The increased detection process of SPT can effectively prevent attackers from bypassing the detection of OTAA. At the same time, the original detection mechanism of OTAA has been retained to ensure the normal use of the join procedure function.

### 3.6. Method for Reducing Power Consumption

LoRaWAN describes a link-based ADR [12] mechanism which can dynamically modify some transmission parameters for the link between ED and GW. To test its performance, we called the FLoRa (Framework for LoRa) in the Omnet simulator. FLoRa is a framework dedicated to simulating LoRa node communication. It supports the Adaptive Data Rate (ADR) to set the data rate and the transmission power of static ED. It is the best way to increase the overall capacity of the network and maximize the battery life of each ED [13]. So we use ADR to optimize performance in every scenario at least. There are kinds of variability in channel quality. By default, ADR selects the maximum snr to establish the best communication connection, but the actual value can be changed to the average value of the snr. To optimize the energy consumption of SPT, we used the ADR+ algorithm in the actual scenario to optimize the energy consumption of packet transmission.

### 3.7. How to Implement SPT

In order to facilitate the demonstration of the experiment, we built a local private sensor network. We also need to make functional modifications to the gateway and server but there is currently no open source about gateway and server for LoRaWAN v1.1. So this requires us to re-implement old version for LoRaWAN protocol. We implemented our mechanism in strict accordance with the OTAA approach in order to ensure the reliability of our experiments. After this process, our demo network can work under the definition from SPT.

## 4. Security Analysis for SPT

In this section, we will demonstrate the reliability and security of SPT in two aspects:

1. Logic Correctness proof by BAN Logic;
2. Formal analysis the SPT by using Scyther.

### 4.1. Logic Correctness Proof Based on BAN Logic

The BAN logic consists of 19 logical rules. For SPT, we will need 4 rules used in this paper.

*1) Rule 1: Nonce Verification Rule*

$$\frac{P|\equiv \#(X),\;P|\equiv Q|∼X\;}{P|\equiv Q|\equiv X}.$$

Expresses that if *P* believes that *X* is fresh and *Q* once said with *X*, then *P* believes that *Q* believes *X*.

*2) Rule 2: Jurisdiction Rule*

$$\frac{P|\equiv Q|\Rightarrow X,\;P|\equiv Q|\equiv X}{P|\equiv X}$$

Expresses that if *P* believes that *Q* has jurisdiction over *X* and *P* trusts *Q* on the truth of *X*, then *P* believes *X*.

*3) Rule 3: Freshness Rule*

$$\frac{P|\equiv \#(X)}{P|\equiv \#(X,Y)}$$

Expresses that if *P* believes that one part *X* of a formula is fresh, then the entire formula (X,Y) must also be fresh.

*4) Rule 4: Belief Rule*

$$\frac{P|\equiv Q|\equiv (X,Y)}{P|\equiv Q|\equiv X}$$

States that if *P* trusts *Q* on the truth of the entire formula (X,Y), then *P* believes that *Q* believes one part *X* of it.

**Establishment of the idealizedprotocol model:**

In order to describe SPT into the BAN Logic, we have removed some irrelevant components. For the generic forms of SPT are provided below:

Message 1: $ED\to NS:\{JoinEUI_{1},DevEUI_{1},DevNonce_{1},MIC_{1}\}$

Message 2: $ED\to NS:\{JoinEUI_{2},DevEUI_{2},DevNonce_{2},MIC_{2}\}$

Message 3: NS→ED:{JoinNonce}

In these models, ED denotes End-Device, NS denotes Network Server.

**Establishment of the initial assumption:**

By analyzing the SPT, some initiative assumptions can be obtained. All of these assumptions are based on default common senses and key agreement verification.

**Hypothesis** **1.** ED∣≡NS∣∼JoinNonce.

**Hypothesis** **2.** $ED\;|\equiv \#(JoinEUI_{1},DevEUI_{1})$.

**Hypothesis** **3.** $NS\;|\equiv \#DevNonce_{1}$.

**Hypothesis** **4.** $NS\;|\equiv (JoinEUI_{1},DevEUI_{1})$.

**Hypothesis** **5.** $NS\;|\equiv ED|\equiv (MIC_{1},MIC_{2})$.

**Establishment of the intended purpose:**

the protocol is secure against various malicious attacks only if the intended purpose is met. In order to complete the authentication proof, the SPT schema must satisfy the following test goals.

Goal 1: $NS\;|\equiv ED|\equiv (JoinEUI_{1},DevEUI_{1})$.

Goal 2: $NS\;|\equiv MIC_{1}$.

Goal 3: $NS\;|\equiv (DevNonce_{1},DevNonce_{2})$.

Goal 4: ED∣≡JoinNonce.

**Protocol analysis:**

Based on the hypotheses and the logical rules we have mentioned, a main procedures of proof can followed like this.

According to the Message 2 and Hypothesis 2, we obtain:

(1) $$NS;|\equiv ED|∼JoinEUI_{1},DevEUI_{1};$$

By Hypothesis 4 and Equation (3), we employ the Rule 1 to derive:

(2) $$NS\;|\equiv ED|\equiv (JoinEUI_{1},DevEUI_{1});$$

which satisfies the **Goal 1**.

By Hypothesis 5 and Message 2, we apply the Rule 2 to deduce:

(3) $$NS\;|\equiv MIC_{1}$$

which satisfies the **Goal 2**.

By Hypothesis 3, we apply the Rule 3 to derive:

(4) $$NS\;|\equiv (DevNonce_{1},DevNonce_{2})$$

which satisfies the **Goal 3**.

By Hypothesis 1 and Message 3, we employ Rule 4 to derive:

(5) ED∣≡NS∣≡JoinNoince

By Equation (7) and Message 2, we can employ Rule 2 to derive:

(6) ED∣≡JoinNonce

which satisfies the **Goal 4**.

By Equation (4), Equation (5), Equation (6) and Equation (8) we demonstrate that the SPT protocol provides secure authentication between the End-Device and Network Server. Therefore, our proposed protocol is logically accurate and is able to guarantee trustworthy communications.

### 4.2. Formal Analysis of SPT and LoRaWAN

Scyther [14] is a tool for the automatic verification of security protocols. It is very useful in analyzing the security vulnerabilities of various communication protocol. It takes as input a security protocol description that includes a specification of intended security properties [15], referred to as security claims, and evaluates these. Scyther assumes that the encryption process is done in an ideal environment. This means that no matter what kind of encryption method(like Data Encryption Standard and Advanced Encryption Standard) is used under this tool, it can be considered as a successful process. In addition, Scyther is mainly used to verify that all logical vulnerability inside the specification. A vulnerability refers to an existence of a way to bypass the normal process and continue with the next steps.

**Formal analysis:**

**Figure 7:**
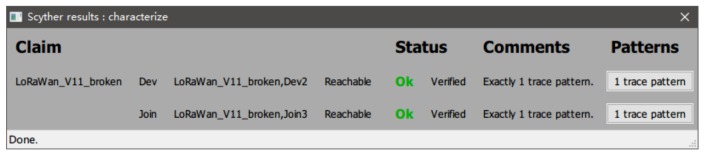
Scyther results characterize the low power wide area network (LoRaWAN).

**Figure 8:**
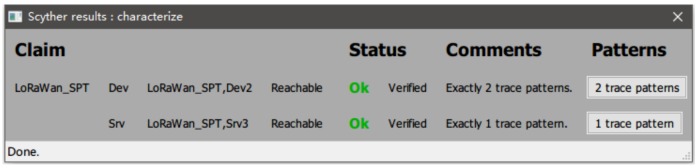
Scyther results characterize the LoRaWAN.

We formalize the join procedure according to the authentication process based on the Mohamed [16]’s work in LoRaWAN v1.1. According to the content of the OTAA, the JoinNonce, JoinEUI, DevNonce and NwkKey are used to create network session long keys: NwkSEncKey, FNwkSIntKey, SNwkSIntKey. The message integrity code (MIC) is consisted of the FNwkSIntKey in the up-link payload. Whereas, the SNwkSIntKey is used for the MIC of down-link data messages. NwkSEncKey and AppSKey keys (network and application) are used for the confidentiality and integrity of the messages exchanged afterward. Finally, JoinNonce, JoinEUI, DevNonce and AppKey are used to create application session long key: AppSKey, the session key shared between the ED and AS and used to encrypt/decrypt application layer payloads. On this basis, we need to add the premise of our setup. When the root key is compromised, the normal node will face the failure to enter the network properly, so we have to add a timeout retransmission step. After formalization, results of characterize pattern from scyther is shown in Figure 7 and Figure 8.

The results summarized in Table 2 show that one claim event: Nisynch is not safe in LoRaWAN V1.1. That means the attacker can use another node to take the place of the normal node. In other sense, the weak relation between join-request and join-accept message for a node is not safe no more. In the sense that if multiple join requests are sending to a server, the replies cannot distinguish between a normal node and a malicious node. The result for LoRaWAN + SPT has no attack events. That means all the behavior we care about is safety. The network server can defend against malicious behaviors.

## 5. Performance Evaluation

In this section, we will test the performance evaluation of SPT in the simulation environment by Omnet++ and a TestBed. In Omnet, we use the FLoRa framework to simulate the communication of LoRa nodes, GW and NS. Communication scenarios and hardware communication parameters such as SF, communication frequency can be set in this framework. We set this experiment in the simulation environment including urban and sub-urban scenarios. There is another advantage of FLoRa is that it can directly collect communication data for each node. We can count the amount of uplink data sent and the amount of downstream data, so we can calculate the delivery ratio by these data. According to the LoRaWAN protocol, performance can be evaluated by measuring the delivery ratio. We measure the power consumption for a real node in the TestBed. A replay attack is also taken in this TestBed to validate the SPT demo.

### 5.1. Delivery Ratio Evaluation

#### 5.1.1. Parameter Setup

We employed FLoRa to evaluate the delivery ratio of urban and sub-urban with ADR. We used the Asia regional parameters for the LoRa physical layer which has shown in in Table 3. The scale for the number of LoRa Node has set from 100 to 700 and each node can initialize itself by picking a random spreading factor and a transmission power level. The value of path loss directly affects the data transfer rate and energy loss, so this factor needs to be taken into account when conducting simulation experiments. In our two scenarios—urban and sub-urban—we have considered three different standard deviations in path loss: an ideal channel, a channel with moderate variability and a channel with typical variability. Parameters for path loss can be checked in Table 4. In the simulation environment, we test whether the communication performance of the SPT will be affected by additional data transmission. At the same time, we also test the normal LoRaWAN as our baseline.

#### 5.1.2. Delivery Ratio Result in Omnet

**1. Ideal environment:** Table 5 has shown the delivery ratio on LoRaWAN and SPT. When there is no variance in the path loss, SPT with ADR algorithm can perform well as the LoRaWAN. The Delivery ratio of the four sets of experiments was maintained between 70% and 80% which can satisfy the requirement.

**2. Moderate variability and typical variability environment:** When moderate variability and typical variability occur in the communication environment, the value of the delivery ratio under the suburban becomes very low, ass seen in Table 6. The main reason for this result is because the ADR algorithm in LoRaWAN calculates the Signal-Noise-Ratio (SNR) based on the maximum value from the received frames. The SNR has decreased the transmission power in the whole network. Therefore, the use of LoRaWAN nodes will result in multiple timeout retransmissions resulting in a decrease in the delivery ratio. ADR in SPT has used the average value of SNR. However, since the SPT itself sends the Njr once more, the probability of packet loss increases correspondingly and finally it is similar to LoRaWAN in the experimental data.

#### 5.1.3. Delivery Ratio Result in Testbed

The delivery ratio of the node in Testbed is better than the result in simulation environment from Table 7. This is because there are fewer interference factors in the real environment. We can also see that the delivery ratio for the node under the two mechanisms is similar. This result can prove that SPT can satisfy the basic communication requirements of the sensor network.

### 5.2. Power Consumption Experiment

#### 5.2.1. Testbed System Setup

**Figure 9:**
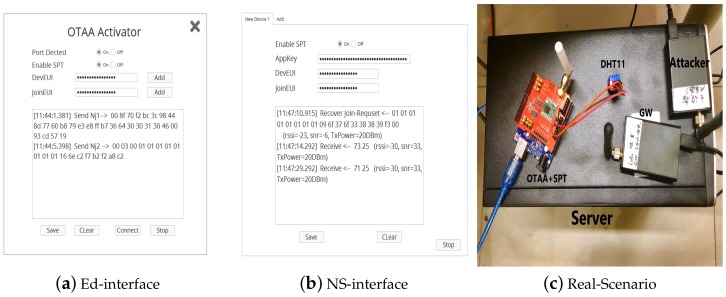
OTAA with SPT UI and Real TestBed.

In order to improve the enhanced security of SPT, we applied it to the Smart Home scenario. Temperature and humidity sensors are commonly used to measure temperature and humidity in a home. Users can make adjustments according to these data. The testbed has shown in Figure 9. The red node is the SX1276 [17] node which connect to a DHT11 Sensor [18] for collecting temperature data. The other two nodes are for attackers and gateway. We develop two interfaces to display the real-time data include payload, RSSI, Snr, txpower. To allow the experimental data to be distinguished, we adjust the actual room temperature values in the different attack tests. We also set up two scenarios of short-range communication and long-distance communication, in which the short-distance communication is about 100 m; the long-distance communication is about 2 km.

#### 5.2.2. Power Consumption Results in Testbed

**Figure 10:**
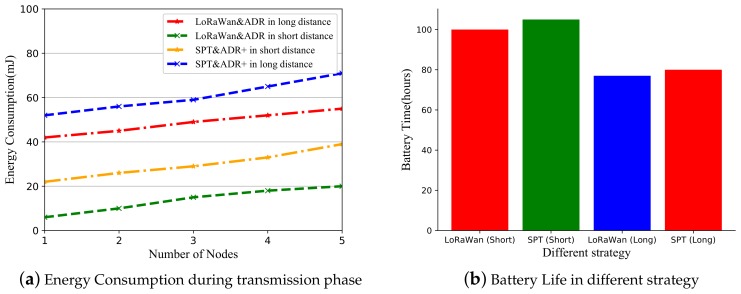
Energy consumption and Battery Life in different distance.

The test environment in the field is close to the ideal environment, so the value of the Delivery ratio is relatively good from Table 7. At the same communication distance, lower SF values can effectively reduce energy consumption. All energy consumption data from Figure 10a is the transmission energy for each node. It shows the energy consumption of LoRaWAN and SPT at different distances. In actual usage scenarios, consuming less energy will effectively extend battery life. Battery life directly determines the normal working hours of a node. In accordance with the specifications of the conventional charging battery, we estimate the usage time of using different strategies according to the operating energy consumption of a node which has shown in Figure 10b. Relying on the ADR+ algorithm, the power of the node will be appropriately reduced, and the battery life will be extended. Although SPT sent more packets than LoRaWAN. But SPT has used the ADR+ algorithm to adjust the spreading factor so that it can decrease some energy. Overall, the energy consumption of SPT is acceptable.

### 5.3. Replay Attack Experiment

#### 5.3.1. Preset Conditions

In our testbed, we let the user’s ED connect to LoRaWAN with the specified protocol. After NS continuously receives DHT11 data for 3 min, user’s ED temporarily goes off-line for 30 s. A replay attack will occur during this time. After the 4th minute, if the temperature data received by the NS is an abnormal temperature value, it means that the replay attack is successful, otherwise, the replay attack fails. We let the red node suffer from attacker in Figure 9. During the attacking phase, the attacker will use different attack models as followed to attack the red ED. Let the ED send data from DHT11 after it finished OTAA or SPT. Then the malicious ED send Abnormal temperature data after it successful launch replay attack. We have set up a total of three forms of attacks, the experimental settings for each attack can be found in the Table 8. Each attack experiment was repeated 15 times and we calculated the average value for all data.

*A* denotes Attack; *Yes* means the attack model can modify it; *No* means attack model does not have the ability to modify it. Attack-1 is used in both scenarios (OTAA and SPT). Attack-1 does not pose a threat to any protocol. The significance of designing this attack model is to prove that the demo works properly. Attack-2 and Attack-3 are actually the same attack model. After the root key is leaked, the attack model will forge a malicious join request to a NS. Attack-2 is for OTAA after attacker has the root key; Attack-3 is only for SPT after attacker has the group data from node.

#### 5.3.2. Attack Results in Testbed

**Figure 11:**
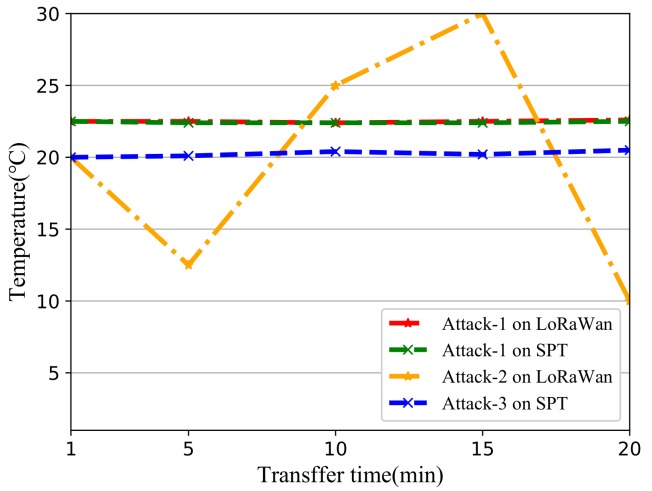
Temperature data under each attack

From Figure 11 we can get all impacts of three attack models on OTAA and SPT. Attack-1 has no effect on either OTAA or SPT. This experiment for Attack-1 can be seen as the baseline of the testbed. From the baseline, we can prove the validity of the firmware we deployed on the testbed. Attack-2 reflected the OTAA was unable to perform any effective defense after the attacker modified the MIC and header with the root key. In the first 5 min of the communication, we let the normal node go off-line once, and let the malicious node perform the replay attack. The malicious node successfully connected to the server and started sending the wrong temperature data, which is why the yellow line is generated. The normal node is not affected by any attacking model when using SPT and the sensor’s temperature data can be correctly transmitted to the server which has been shown in Attack-3. We cannot see any difference from the temperature data on the surface but the server successfully resisted the attack by conducting an attack experiment.

## 6. Conclusions

In this paper, We have designed a new system model named SPT to detect and defend against possible replay attacks under the assumption that the root key is compromised. From the perspective of theoretical certification, we prove the effectiveness and feasibility of OTP in SPT. Sending join-request packets can effectively deceive the attacker and prevent the eavesdropper from imitating this format. The proposed SPT has been evaluated by using Ban-logic, Scyther, Omnet++ and the implementation of a testbed. The test results prove the feasibility and effectiveness of our solution. The optimized ADR algorithm is used to offset some power consumption in the SPT implementation in the testbed. The algorithm can effectively reduce part of the extra energy consumption to make it is reasonable in the real world. With the help of SPT, the Network Server has the ability to defend against malicious behavior from attackers.

## 7. Related Work

There are many researchers in the wireless sensor network who have done security research. Qiu et al. [19] proposed an optimization strategy for the structure of large-scale wireless sensor networks to avoid cyber attack. Geneiatakis et al. [3] analyze and sort out the security issues of smart homes according to the type of security attacks. They point out that the main reason for the security problem is that the existing protocols have defects, which can make the IoT devices available to eavesdroppers [20,21] present their research about security risks in the smart home automation system. Their analysis results were organized in five categories including software, hardware, information, communication and human-being reasons. Their research also pointed out that the most serious causes of various security risks are caused by firmware software API calls and incorrect permission control.

Research on the LoRaWAN protocol can be made in two parts. For the first part, some researchers have proposed the security flaws in the protocol through theoretical analysis and experimental verification. Tomasin and Aras [22] have identified vulnerabilities in LoRaWAN. They used Semtech’s nodes to verify the randomness of DevNonce, proving that its randomness would cause the failure of normal nodes to enter the network and the feasibility of replay attacks. Their work proves that it is possible to launch replay attacks on the existing LoRaWAN protocol.For the another part, many researchers have proposed strategies for detecting and preventing replay attacks based on the existing LoRaWAN protocol. Jae [23] proposed a method that under the existing protocol, user can use different formats of join request to join the network. Their point is good; the most effective way to solve the problem is to protect join request data. However, because the logic is too simple, the attacker can grasp the rule of the method by intercepting the data several times. Seung Jae, who also changed the join-request, proposed [24] a token-based authentication method so that the join-request will not be sent directly to the gateway, but will continue using the token to calculate the original incoming data. This method has a very limited defense effect and imposes a heavy load on the server. Tahsin [25] proposed using different parameters to calculate the AppSKey in each communication process to enhance the randomness of the key to resist the replay attack. This method can work, but once the attacker makes multiple replay attacks to the same ED, the AppSKey held by the attacker will always be correct sooner or later.

Some researchers have focused their research on the protocol communication processes and key management in various protocols or services. They tried to redefine a new LoRa communication protocol to completely circumvent the flaws in the original agreement. Miller [26] analyzed possible attacks on LoRaWAN, and asserted that the encryption key generation process and key management policy can be enhanced. He thinks that all end-devices, gateways and servers should have their own user verification and key protection policies, so that it can guarantee the communication security. Naoui et al proposed a new security architecture for LoRaWAN. It uses proxy nodes to perform partial functions of GW. These nodes evaluate the reliability of neighboring nodes and then create a reliability environment which can send messages to all EDs. With this method, each ED can select an available proxy node with the highest reliability to transmit its data. Qiu et al. [27] proposed a spider-web-like transmission mechanism in the vehicular Ad Hoc Networks. Their work has improved the packet delivery ratio and average transmission delay of emergency data. Bouguera [28] proposed some countermeasures to enhance the security level of LoRaWAN with complex operations.This method also increases the power consumed by the data encryption, data decryption and so on. In [29], some key management with new low power protocol has been described. The protocol can circumvent some vulnerabilities in LoRaWAN, but they do not have good compatibility with the LoRa’s server. Therefore, the practical value is affected.

## Figures and Tables

**Table 1:** Abbreviation.

Notations	Description
*ED*	End Device same as node
*GW*	Gate Way between Node and Network Server
*NS*	NetWork Server
*jr*	Join request
*Njr*	New Join request
*en-*	encode some message
*snr*	signal-to-noise ratio
*ae*	aes128-encryption method

**Table 2:** LoRaWAN V1.1 and SPT Scyther validation results.

Protocol	LoRaWAN v1.1		LoRaWAN+SPT	
**Claim**	**Status**	**Attack Patterns**	**Status**	**Attack Patterns**
Role:Node				
Alive	Ok	No attack	Ok	No attack
Weakagree	Ok	No attack	Ok	No attack
Niagree	Ok	No attack	Ok	No attack
Nisynch	Fail	**At least 1 attack**	Ok	No attack
SKRAPPSKey	Ok	No attack	Ok	No attack
SKRNwkSKey	Ok	No attack	Ok	No attack
SKRSnwkSIntKey	Ok	No attack	Ok	No attack
SKRNwkSencKey	Ok	No attack	Ok	No attack
SKRJSEncKey	Ok	No attack	Ok	No attack
SKRJSIntKey	Ok	No attack	Ok	No attack
Role:Server				
Alive	Ok	No attack	Ok	No attack
Weakagree	Ok	No attack	Ok	No attack
SKRAPPSKey	Ok	No attack	Ok	No attack
SKRNwkSKey	Ok	No attack	Ok	No attack
SKRSnwkSIntKey	Ok	No attack	Ok	No attack
SKRNwkSencKey	Ok	No attack	Ok	No attack
SKRJSEncKey	Ok	No attack	Ok	No attack
SKRJSIntKey	Ok	No attack	Ok	No attack

**Table 3:** The simulation parameters.

Parameter	Value
Carrier Frequency	433 MHz
Bandwidth	125 kHz
Code Rate	3/4
Spreading Factor	7 to 12
Transmission Power	2 dBm to 14 dBm

**Table 4:** Standard deviation of the path loss.

Scenario	Ideal	Moderate Variability	Typical Variability
Urbany	0	1.78	3.55
Sub-urban	0	3.56	7.2

**Table 5:** Delivery Ratio in ideal simulation environment for over-the-air-activation (OTAA) and SPT.

Node	OTAA-u	OTAA-su	SPT-u	SPT-su
100	99	85	**99%**	**83%**
200	95	82	**97%**	**82%**
300	92	87	**96%**	**87%**
400	93	83	**95%**	**80%**
500	98	84	**91%**	**85%**
600	92	82	**90%**	**83%**
700	90	81	**92%**	**82%**

**Table 6:** Delivery Ratio in moderate variability and Typical variability in Omnet for OTAA and SPT.

Nodes	Moderate Variability				Typical Variability			
	OTAA -Urban	OTAA -Suburban	SPT -Urban	SPT -Suburban	OTAA -Urban	OTAA -Suburban	SPT -Urban	SPT -Suburban
100	65	22	**64%**	**23%**	23	16	**22%**	**17%**
200	66	23	**63%**	**22%**	22	17	**21%**	**16%**
300	63	23	**64%**	**24%**	24	17	**20%**	**15%**
400	62	24	**65%**	**25%**	25	16	**24%**	**15%**
500	64	26	**63%**	**26%**	21	18	**22%**	**14%**
600	65	22	**62%**	**21%**	20	17	**23%**	**15%**
700	61	23	**63%**	**22%**	20	16	**25%**	**13%**

**Table 7:** Delivery Ratio in Test Bed for OTAA and SPT in different distances.

Node	OTAA-Short	OTAA-Long	SPT-Short	SPT-Long
**1**	98	82	**97%**	**83%**
**2**	95	84	**96%**	**84%**
**3**	92	85	**95%**	**85%**
**4**	95	86	**94%**	**85%**
**5**	96	86	**93%**	**86%**

**Table 8:** Configuration for each attack model.

	MHDR	Unique Part	DevNonce	MIC	Temperature	Distance
**A-1**	No	No	Yes	No	22 ${}^{\circ }$C	0.1 km/2 km
**A-2**	Yes	No	Yes	Yes	20 ${}^{\circ }$C	0.1 km/2 km
**A-3**	Yes	No	Yes	Yes	20 ${}^{\circ }$C	0.1 km/2 km
